# Enhancement of Alkaline Protease Activity and Stability via Covalent Immobilization onto Hollow Core-Mesoporous Shell Silica Nanospheres

**DOI:** 10.3390/ijms17020184

**Published:** 2016-01-29

**Authors:** Abdelnasser Salah Shebl Ibrahim, Ali A. Al-Salamah, Ahmed M. El-Toni, Khalid S. Almaary, Mohamed A. El-Tayeb, Yahya B. Elbadawi, Garabed Antranikian

**Affiliations:** 1 Department of Botany and Microbiology, College of Science, King Saud University, Riyadh 11451, Saudi Arabia; aaasalah98@yahoo.com (A.A.A.-S.); kalmaary@ksu.edu.sa (K.S.A.); mohamedmicro32@yahoo.com (M.A.E.-T.); alzeeem95@yahoo.com (Y.B.E.); 2 Department of Chemistry of Natural and Microbial Products, Pharmaceutical Industries Research Division, National Research Center, El-Buhouth St., Dokki, Cairo 12311, Egypt; 3 King Abdullah Institute for Nanotechnology, King Saud University, Riyadh 11451, Saudi Arabia; aamohammad@ksu.edu.sa; 4 Central Metallurgical Research and Development Institute, Helwan, Cairo 11421, Egypt; 5 Institute of Technical Microbiology, Hamburg University of Technology, Hamburg 21073, Germany; antranikian@tuhh.de

**Keywords:** alkaline protease, immobilization, hollow core-mesoporous shell silica nanospheres, nanotechnology, alkaliphiles, detergents

## Abstract

The stability and reusability of soluble enzymes are of major concerns, which limit their industrial applications. Herein, alkaline protease from *Bacillus* sp. NPST-AK15 was immobilized onto hollow core-mesoporous shell silica (HCMSS) nanospheres. Subsequently, the properties of immobilized proteases were evaluated. Non-, ethane- and amino-functionalized HCMSS nanospheres were synthesized and characterized. NPST-AK15 was immobilized onto the synthesized nano-supports by physical and covalent immobilization approaches. However, protease immobilization by covalent attachment onto the activated HCMSS–NH_2_ nanospheres showed highest immobilization yield (75.6%) and loading capacity (88.1 μg protein/mg carrier) and was applied in the further studies. In comparison to free enzyme, the covalently immobilized protease exhibited a slight shift in the optimal pH from 10.5 to 11.0, respectively. The optimum temperature for catalytic activity of both free and immobilized enzyme was seen at 60 °C. However, while the free enzyme was completely inactivated when treated at 60 °C for 1 h the immobilized enzyme still retained 63.6% of its initial activity. The immobilized protease showed higher *V_max_*, *k_cat_* and *k_cat_*/*K_m_*, than soluble enzyme by 1.6-, 1.6- and 2.4-fold, respectively. In addition, the immobilized protease affinity to the substrate increased by about 1.5-fold. Furthermore, the enzyme stability in various organic solvents was significantly enhanced upon immobilization. Interestingly, the immobilized enzyme exhibited much higher stability in several commercial detergents including OMO, Tide, Ariel, Bonux and Xra by up to 5.2-fold. Finally, the immobilized protease maintained significant catalytic efficiency for twelve consecutive reaction cycles. These results suggest the effectiveness of the developed nanobiocatalyst as a candidate for detergent formulation and peptide synthesis in non-aqueous media.

## 1. Introduction

Currently enzymes are widely used as alternative of chemical catalysts in various industries; and playing a significant role in development of ecofriendly technology, owing to their unique properties including high specificity, mild reaction conditions, low toxicity and selectivity [1,2]. Proteases (EC 3.4.21-24 and 99) represent one of the most important classes of industrial enzymes, that account for up to 60% of the total enzyme sales worldwide [3]. Proteases have long been used to catalyze the hydrolysis of various proteins in aqueous solutions, in addition to peptides synthesis in non-aqueous media [4]. Alkaline proteases, accounting alone for approximately 40% of the total global enzymes market, proved particularly suitable for several industrial applications such as detergent, pharmaceutical, leather, dairy, silk, soy processing, brewery, meat tenderization, and waste management [5,6]. This is attributed mainly to significant activity and operational stability under harsh operational conditions, including high pH and temperature and in the presence of surfactants [4,7,8].

Enzyme immobilization on solid supports represents an efficient approach to improve enzyme stability as well as offering unique merits over the soluble biocatalyst such as operational stability, reusability of the enzyme, bioprocess control, and simplifying product separation [9,10]. Recent advance in nanotechnology have provided diverse nanostructured materials that are more effective for biocatalyst immobilization. It was shown that nanostructured materials can enhance the efficiency of the immobilized enzymes by providing the maximum limits in balancing the key parameters that determine the effectiveness of immobilized enzymes such as large surface area, minimum mass transfer resistance, and high enzyme loading [11]. However, selection of suitable enzyme carriers and the applied immobilization approach is of significant importance for successful immobilization processes. Among inorganic materials, mesoporous silica nanoparticles (MPS) are of special interest for enzyme immobilization technology, which is characterized by large surface areas, easily functionalizable surfaces and confined nanospace for enzyme inclusion, in addition to high biocompatibility and low toxicity [12]. MPS nanoparticles with different morphologies have been fabricated and applied in enzyme immobilization [13,14]. However, hollow mesoporous silica nanoparticles are characterized by several unique features that make them promising candidates for biocatalyst immobilization such as inner voids suitable for inclusion of proteins with various sizes, having surfaces (inner and outer) that can be easily chemically modified and showing high diffusion rate within the mesostructure of the silica shells [15].

In the present study, a recently characterized alkaline protease from alkaliphilic *Bacillus* sp. NPST-AK15 [16,17] was immobilized onto hollow core-mesoporous shell silica (HCMSS) nanospheres by two approaches including covalent and physical immobilization. Subsequently, the properties of immobilized protease were investigated.

## 2. Results and Discussion

### 2.1. Fabrication and Characterization of Hollow Core-Mesoporous Shell Silica Nanospheres

**Figure 1:**
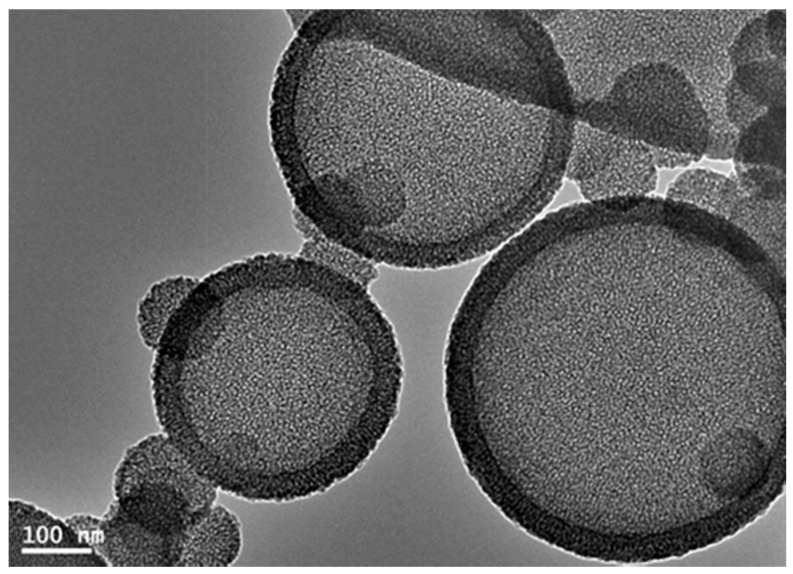
TEM images of amino-functionalized hollow core-mesoporous silica (HCMSS–NH_2_) nanospheres prepared by anionic surfactant.

Hollow core-mesoporous shell silica (HCMSS) nanospheres were synthesized by anionic surfactant through a soft-templating route assisted by ultrasonic waves. In this route, negatively charged silica nuclei are reacted with anionic surfactant through a co-structure directing agent (3-aminopropyltrimethoxysilane (APMS)) to produce mesoporous silica nanospheres. On the other hand, hollow core structures are obtained through assembling of the anionic surfactant micelles on the bubbles created in the solution by ultrasonic waves and then the silica nuclei are precipitated to form a hollow core structure. The template was removed using solvent extraction to maintain the functionalization of HCMSS spheres with amino groups. Figure 1 shows amino functionalized hollow core-mesoporous shell silica spheres (HCMSS–NH_2_) using anionic surfactant. HCMSS–NH_2_ possessed spheres with sizes ranged from 200–300 nm while the shell thickness was around 30 nm. The non-functionalized hollow core-mesoporous shell spheres (HCMSS-non) were produced by calcination at 550 °C of amino-functionalized ones. Ethane functionalization was conducted on HCMSS-non spheres through post-synthesis approach. Both calcination and ethane functionalization steps did not affect the morphology of hollow core nanostrucures.

**Figure 2:**
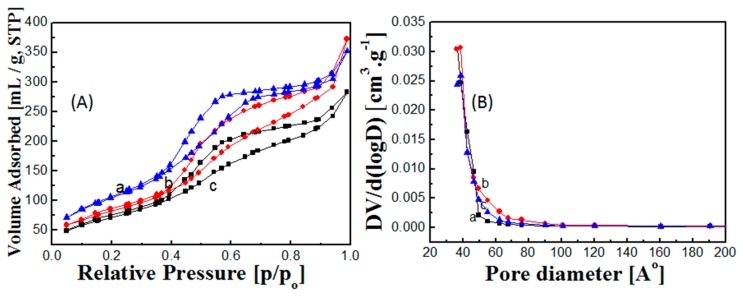
(**A**) N_2_ adsorption-desorption isotherms and (**B**) pore size distribution of (a) non-functionalized, (b) amino-functionalized and (c) ethane-functionalized of hollow core-mesoporous shell silica nanospheres.

The typical nitrogen adsorption/desorption isotherms for the hollow core-mesoporous shell silica sphere samples with various functionalities are presented in Figure 2A. The isotherms showed the type IV curves that depicted the uniform mesoporous feature of hollow core-mesoporous shell silica nanospheres [18]. The Brunauer-Emmett-Teller (BET) surface area and total pore volume of HCMSS–NH_2_ nanospheres were 307.13 cm^2^·g^−1^ and 0.576 cc·g^−1^, respectively. Removal of amino groups by calcination resulted in increment of surface area to 394.27 cm^2^·g^−1^ while the pore volume was almost closer to amino-functionalized sample (0.545 cc·g^−1^). These results are expected because the calcination would remove the amino groups which lead to improvement of surface area of hollow core nanostructures. Ethane functionalization through post-synthesis approach resulted in decrease of both surface area and pore volume to 270.42 cm^2^·g^−1^ and 0.438 cc·g^−1^, respectively. This is due to blockage of surface sites and mesopores with ethane groups. Pore size distribution for the hollow core-mesoporous shell silica sphere samples with different functionalities are illustrated in Figure 2B. All samples showed an identical pore size distribution profile, which centered around 3.6 nm. In that regard, reduction of the total pore volume upon functionalization without reduction of the mesopores size suggested that the ethane groups were concentrated on the bottom or the entrance of mesopores rather than homogenously distributed all over the channels and therefore the pore volume was reduced while pore size was not affected.

**Figure 3:**
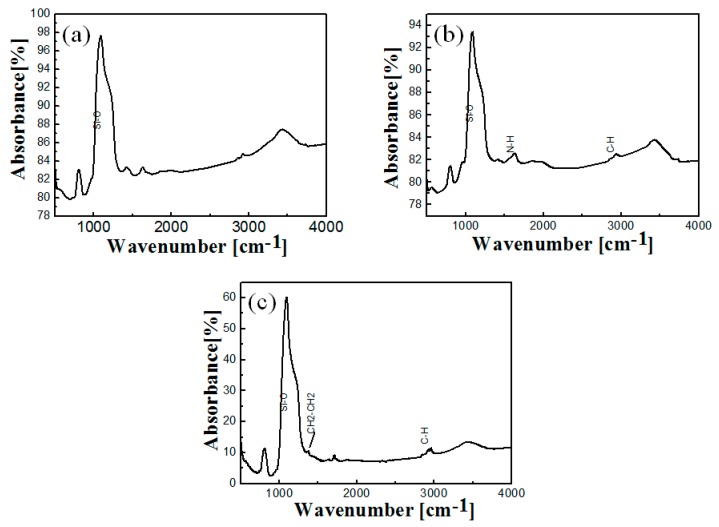
FT-IR spectra of (**a**) non-functionalized; (**b**) amino-functionalized and (**c**) ethane-functionalized hollow core-mesoporous shell silica nanospheres.

FT-IR measurements were made and demonstrated that different functional groups existed in the hollow structure as shown in Figure 3. Non-functionalized hollow silica spheres (Figure 3a) showed main Si–O peak which is characteristic for silica at 1050–1250 cm^−1^. Amino functionalization of hollow nanospheres (Figure 3b) through using APMS can be confirmed from the presence of a N–H peak at 1637 cm^−1^ [19]. Functionalization of hollow silica spheres with ethane group (CH_2_–CH_2_) was shown by the appearance of a peak at 1415 cm^−1^ together with a C–H stretching vibrations peak at 2862 cm^−1^ which confirmed the presence of hydrophobic ethane groups within mesochannels as shown in Figure 3c [20]. However, the appearance of an N–H peak at 1637 cm^−1^ and C–H at 2862 cm^−1^ (from APMS functionalization) as seen in Figure 3a (non-functionalized) could indicate the presence of some residual amino and ethoxy groups from APMS functionlization that were not removed completely during the calcination process. However, weak intensity of N–H and C–H peaks in Figure 3a suggest low content of these functional moieties due to the effect of heat treatment.

### 2.2. Protease Immobilization

Purification of alkaline protease from alkaliphilic *Bacillus* sp. NPST-AK15 culture supernatant was carried out through a combination of ammonium sulfate precipitation and anion exchange chromatography as we described in a previous work [17]. The purified enzyme was immobilized onto the synthesized HCMSS nanospheres by covalent attachment and physical adsorption. Three samples were used for physical immobilization that included HCMSS-non, HCMSS–C_2_H_5_, and HCMSS–NH_2_. For covalent immobilization, the amino functionalized hollow core-mesoporous silica (HCMSS–NH_2_) nanospheres were first activated by glutaraldehyde as a bifunctional cross linking agent prior to protease conjugation. The crosslinking of the matrix with glutaraldehyde takes place in two steps in which the free amino groups within the mesopores and hollow core of HCMSS–NH_2_ nanospheres react with terminal aldehyde groups of glutaraldehyde molecules to form a Schiff-base linkage and then the free amino groups in protease molecules condense with the other free terminal aldehyde groups of glutaraldehyde to form a second Schiff-base bonding [21]. The results presented in Table 1 revealed that NPST-AK15 protease was immobilized successfully onto different hollow core-mesoporous shell silica (HCMSS) nanospheres either by covalent or physical immobilization. However, immobilization of NPST-AK15 protease by covalent conjugation was more effective exhibiting much higher immobilization yield (75.6%) and loading efficiency (73.6%) relative to physical immobilization, which can be attributed to the strong covalent bonds formed between the protease molecules and the activated HCMSS–NH_2_ nanospheres through the cross linking agent. On the other hand, immobilization of NPST-AK15 protease by physical adsorption gave low immobilization yield and loading efficiency in all tested supports, suggesting weak electrostatic and hydrophobic interaction between the NPST-AK15 protease molecules and HCMSS nanospheres [22].

**Table 1:** Immobilization of NPST-AK15 alkaline protease onto hollow core-mesoporous shell silica (HCMSS) nanospheres. The standard deviations were in the range of 1%–3%.

Matrix	Immobilization Method	Immobilization Yield * (%)	Activity Yield ** (%)	Loading Efficiency *** (%)
HCMSS-non	Physical adsorption	44.6	40.0	41.2
HCMSS–NH_2_	Physical adsorption	47.3	42.3	44.7
HCMSS–C_2_H_5_	Physical adsorption	19.3	16.3	18.0
HCMSS–NH_2_	Covalent attachment	75.6	72.2	73.6

* Immobilization yield (%) = [(A − B)/A] × 100, where A is the total activity of the enzyme added in the initial immobilization solution and B is the activity of the unbound protease; ** Activity yield = (C/A) × 100, where A is the total activity of the enzyme added in the initial immobilization solution, and C is activity of the immobilization protease; *** Loading efficiency = [(P_i_ − P_unb_)/P_i_] × 100, where P_i_ and P_unb_ are the initial protein subjected to immobilization, and the unbound protein, respectively.

### 2.3. FT-IR of Free and Immobilized Protease

**Figure 4:**
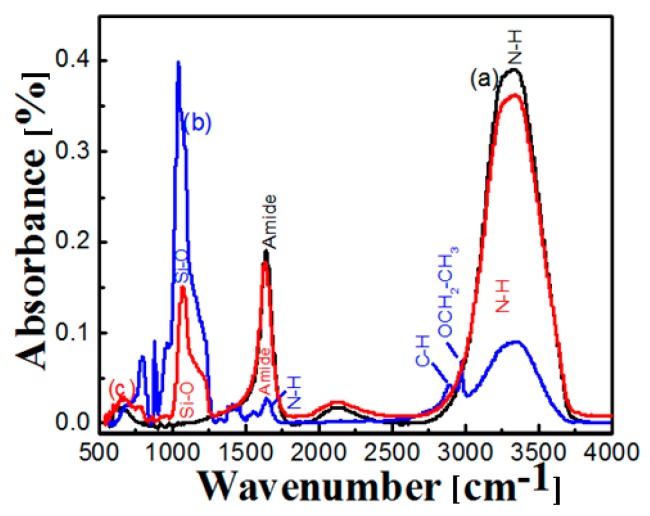
FTIR spectra of (a) Free NPST-AK15 protease enzyme (balck); (b) amino-functionalized hollow core-mesoporous silica (HCMSS–NH_2_) nanospheres (blue) and (c) NPST-AK15 protease immobilized onto amino-functionalized hollow core-mesoporous silica (HCMSS–NH_2_) nanospheres (red).

The binding of the NPST-AK15 protease to HCMSS–NH_2_ was further confirmed by FT-IR analysis. FT-IR spectra for pure free NPST-AK15 protease, HCMSS–NH_2_ nanospheres and immobilized enzyme are illustrated in Figure 4. The spectrum of protease (Figure 4a) showed a strong peak at range of 1640–1650 cm^−1^ which corresponds to the amide I and amide II groups [10]. The strong broad band appearing at 3100–3600 cm^−1^ can be attributed to N–H stretching of protease amides [10]. FT-IR spectrum of HCMSS–NH_2_ nanospheres is shown in Figure 4b. The strong peak at 1050–1250 cm^−1^ is attributed to Si–O bonding [19]. The amino functionalization of HCMSS spheres was demonstrated from the appearance of an N–H, peak at 1637 cm^−1^ as well as OCH_2_CH_3_ and C–H stretching vibrations peaks at 2930 and 2862 cm^−1^, respectively [19]. The FT-IR of the immobilized protease shown in Figure 4c indicated the presence of a strong peak at 1640–1650 cm^−1^ for amide, and a broad one at 3100–3600 cm^−1^ for the N–H group of the protease, together with the main peaks of hollow core-mesoporous shell silica spheres as Si–O bond at 1050–1250 cm^−1^. These results demonstrate and confirm successful immobilization of the NPST-AK15 protease onto HCMSS–NH_2_ nanospheres.

### 2.4. Loading Capacity

**Figure 5:**
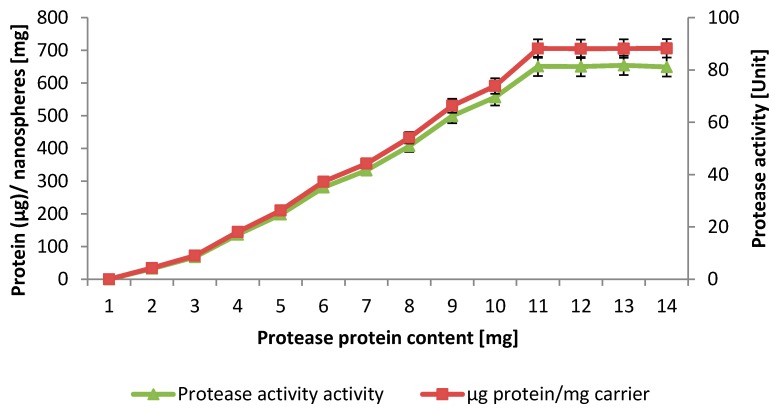
Loading efficiency of hollow core-mesoporous shell silica nanospheres for immobilization of NPST-AK15 protease.

The loading capacity of Ac–HCMSS–NH_2_ nanospheres was estimated by covalent immobilization of various amounts of NPST-AK15 protease and ranged from 50 to 200 μg protein/10 mg nanoparticles. The results shown in Figure 5 indicated that the amount of loaded protease increased with increasing enzyme concentration giving maximum loading of 88.1 μg protein/mg carrier, and then remained constant. This result shows that Ac–HCMSS–NH_2_–NH_2_ nanospheres have high loading capacity for covalent immobilization of NPST-AK15 protease [23,24,25], which can be attributed to the high surface area (307.13 cm^2^·g^−1^) and pore volume of the nanoparticles (0.576 cc·g^−1^, respectively) available for the enzyme conjugation. Furthermore, functionalization of HCMSS nanospheres with amino groups through *in situ* approach rather than *post*-*synthesis* method resulted in integration of a higher density of amino groups available for protease conjugation [26,27]. Thus, a protein loading of 88.1 µg protein per mg Ac–HCMSS–NH_2_–NH_2_ nanospheres was used in all subsequent investigations.

### 2.5. Properties of the Immobilized NPST-AK15 Protease

#### 2.5.1. Influence of pH and Temperature

**Figure 6:**
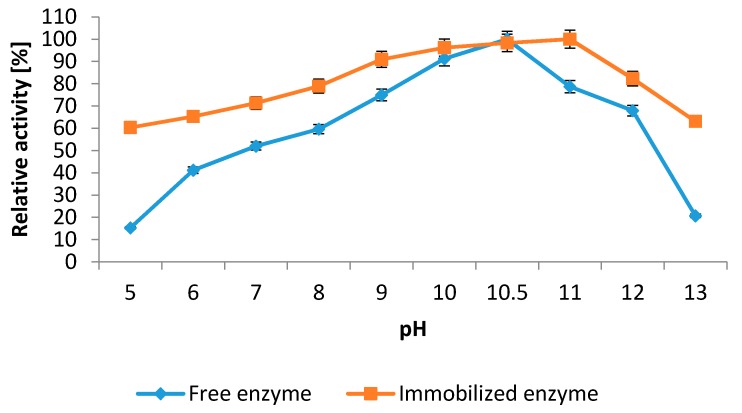
Effect of pH on the activity of the free and immobilized NPST-AK15 protease. Enzyme activity was measured at 55 °C. The results represent the mean of three separate experiments, and error bars are indicated.

The results of the effect of pH on the catalytic activity of both free and immobilized NPST-AK15 protease show that the catalytic activity was increasing as pH increased, reaching maximum activity at pH 10.5 and 11.0 for soluble and immobilized protease, respectively. Hence, the optimum pH of alkaline protease immobilized on Ac–HCMSS–NH_2_ nanospheres was shifted toward the alkaline range by 0.5 units in comparison with the free protease (Figure 6). In addition there was significant increment of the relative activities of the immobilized NPST-AK15 protease at a wide pH range (pH 5–13) in comparison with free enzyme. The slight shift of optimal pH value to the alkaline region was probably due to the change of its microenvironment caused by the immobilization of the enzyme on the carrier. An increase of the optimum pH was observed for the other enzymes immobilized on the solid matrix [25,28].

**Figure 7:**
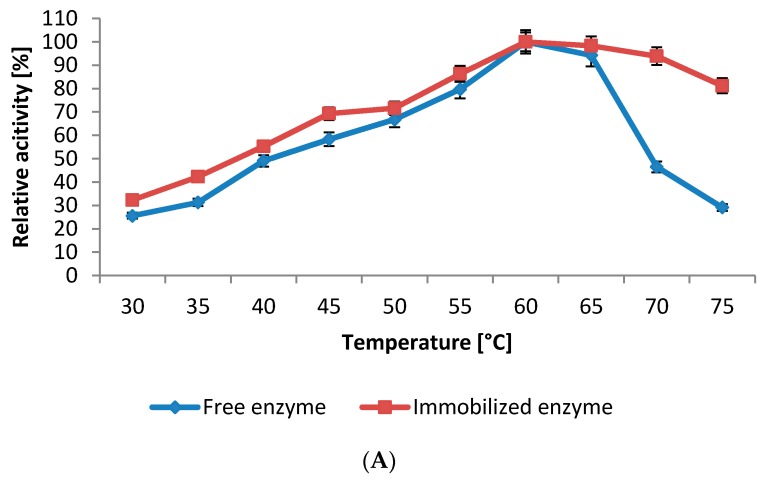

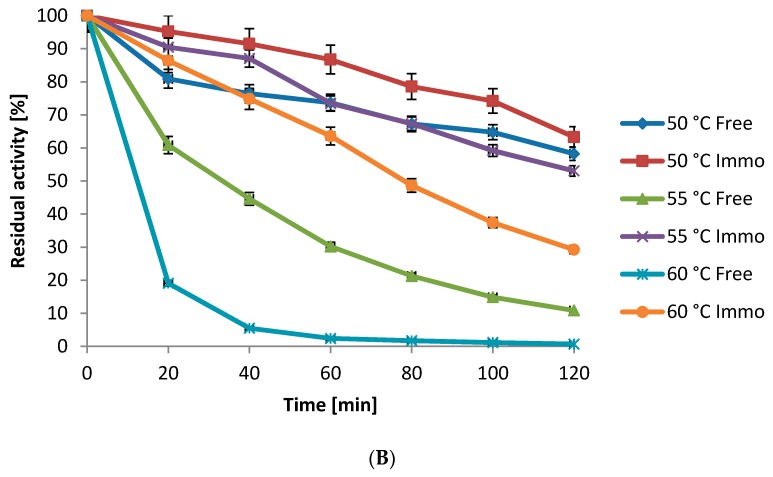
Effect of temperature on activity (**A**) and stability (**B**) of free and immobilized NPST-AK15 protease. Protease activity was measured under the standard assay conditions at various temperatures (30–75 °C) at pH 10. For thermal stability, the enzyme was pre-incubated at different temperatures for 2 h at pH 11.0, and the residual enzyme activities were estimated at regular intervals under standard assay conditions. The non-heated enzymes were taken as 100%. The results represent the mean of three separate experiments, and error bars are indicated.

The results of the influence of reaction temperature on the catalytic activity of soluble and immobilized enzyme revealed that the optimum temperature for both free and immobilized enzyme was 60 °C (Figure 7A). However, the immobilized protease exhibited higher relative activities in comparison with soluble enzyme, especially at high temperatures (65–75 °C). In addition, the thermal stability of free and immobilized protease was investigated at various temperatures (50–60 °C). As shown in Figure 7B the protease immobilized onto the nanosphere showed much better thermal stability compared to free enzyme. After treatment for 1 h at 55 °C, the immobilized enzyme still retained 73.5% of its initial activity, while the free protease maintained only 30%. Moreover, while the soluble enzyme was completely inactivated when treated for 1 h at 60 °C the immobilized enzyme still retained 63.6% and 29.3% of its initial activity, after treatment for 1 and 2 h, respectively. The significant improvement of the immobilized NPST-AK15 protease thermal stability is mostly attributed to multipoint covalent attachment of protease molecules to the matrix that protect the protease tertiary structure and prevent conformation transition of the enzyme upon heating, leading to an improved thermal stability [9,29].

#### 2.5.2. Kinetics Studies

**Figure 8:**
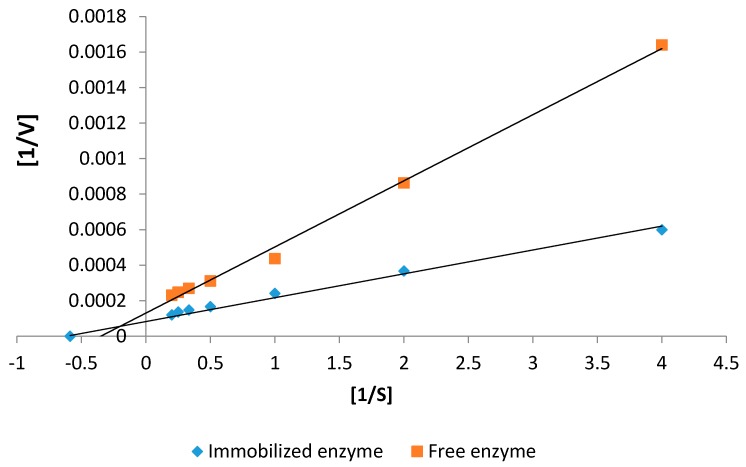
Estimation of kinetic parameters of the free and immobilized NPST-AK15 protease. The enzyme activity was measured at various casein concentrations (1.0–10.0 mg/mL) at pH 11 and 60 °C. The *K*_m_ and *V*_max_ values were determined using linearized Lineweaver-Burk plot. S: Substrate concentration; V: Protease specific activity.

Investigation of the kinetic parameters (*K*_m_ and *V*_max_) is also a critical feature for investigating the effectiveness of an immobilization process. Kinetics parameters of both free and immobilized protease were estimated using Lineweaver–Burk plot by using casein as a substrate at pH 11 and 60 °C. The Michaelis–Menten constant (*K*_m_) of free and immobilized NPST-AK15 protease were 0.109 and 0.074 mM, respectively, which clearly indicated increase of the enzyme affinity toward the substrate by about 1.5-fold up on immobilization onto Ac–HCMSS–NH_2_ nanospheres (Figure 8; Table 2). This is mostly due to high mass transport of reactants and products within the carrier which is a unique feature of the nanostructure materials. Also the Brownian motion of the nanospheres could result in enhanced substrate-enzyme interaction [30]. This behavior of protease immobilized onto Ac–HCMSS–NH_2_ nanospheres was superior to several nanoparticles bound to enzyme [25,31]. In addition, the maximum activity (*V*_max_), enzyme turnover number (*k*_cat_), and catalytic efficiency (*k*_cat_/*K*_m_) values of NPST-AK15 protease were increased by about 1.6-, 1.6-, and 2.4-fold, respectively, upon immobilization on to HCMSS–NH_2_ nanospheres. This may be due to more efficient conformation of immobilized protease within the nanoscale spaces of the hollow nanospheres in respect to free enzyme [28]. The high affinity toward substrate and catalytic activity of NPST-AK15 alkaline protease immobilized onto Ac–HCMSS–NH_2_ nanospheres indicate the efficiency and effectiveness of applied support and immobilization approach.

**Table 2:** Kinetic parameters of free and immobilized NPST-AK15 alkaline protease. The standard deviations were in the range of 1.5%–3.1%.

Kinetic Parameters	Free Protease	Immobilized Protease
*K*_m_ (mM)	0.109	0.074
*V*_max_ (µM·min^−1^·mg^−1^)	41.2	66.1
*k*_ca__t_ (s^−1^)	1156.5	1855.4
*k*_cat_/*K*_m_ (mM^−^^1^·s^−^^1^)	10.6 × 10^3^	25.1 × 10^3^

#### 2.5.3. Effect of Inhibitors and Organic Solvents

The results of the influence of some inhibitors on catalytic activity of free and immobilized NPST-AK15 protease are summarized in Table 3. Phenylmethanesulfonyl fluoride (PMSF) completely inhibited the free protease activity at 5.0 mM, suggesting it belongs to serine protease family [3,17]. However, the immobilized enzyme retained 86.3% of its initial activity. In addition, while the free protease lost about 75% of its initial activity after treatment with 5 mM dithiothreitol (DTT), the immobilized protease maintained 97.5% at the same conditions. The metal chelating agent, EDTA, caused inhibition of the soluble enzyme to the extent of 60.2% and 30.5% at concentrations of 2 and 5.0 mM, respectively, suggesting that some metals may play a role in the activity/stability of the enzyme [17], whereas at the same conditions the immobilized protease still retained 95% and 81.5% of its initial values, respectively. However, there was no significant difference between the effect of β-mercaptoethanol (β-ME) on free and immobilized NPST-AK15 protease. Protection of protease against such inhibitors upon immobilization onto HCMSS–NH_2_ nanospheres could be explained in two different ways: (i) In case of allosteric inhibitor in which it interacts with the enzyme at a site other than the catalytic center, it is likely immobilization of the enzyme through that niche leads to distortion of the allosteric site and subsequently causes significant reduction of the inhibition; (ii) Alternatively, if the inhibitor interacts directly with the active center, the process of enzyme immobilization may cause a slight change in the enzyme active site that affects the inhibitor binding to enzyme with less effect on the substrate-enzyme binding especially in the case of high molecular weight substrates [9].

**Figure 9:**
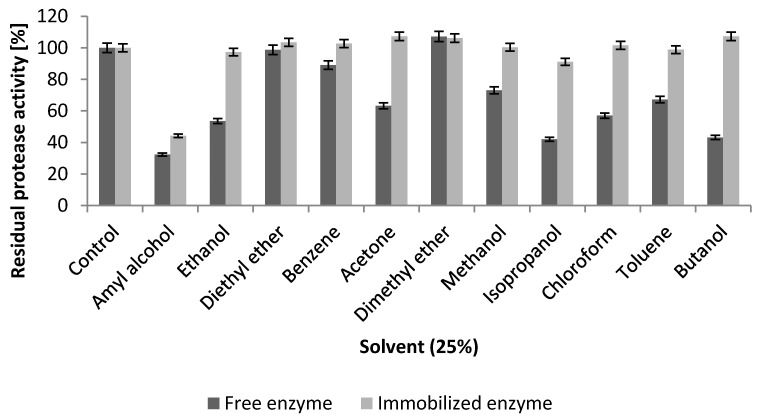
Effect of organic solvents on stability of free and immobilized NPST-AK15 protease.

As shown in Figure 9, the stability of the NPST-AK15 protease in various organic solvents was greatly improved upon covalent immobilization onto the Ac–HCMSS–NH_2_ nanospheres. The results revealed that the stability of the immobilized protease in butanol, isopropanol, chloroform and ethanol was significantly increased by about 2.5-, 2.2-, 1.8- and 1.8-fold respectively, in respect to the free enzyme. In addition, its stability in methanol, toluene, and chloroform was increased by about 1.1-fold. in acetone, toluene, methanol, amyl alcohol and methanol by about 1.7-, 1.5-, 1.4-, and 1.4-fold, in comparison to free enzyme, respectively (Figure 9). The significant enhancement of stability in solvent for NPST-AK15 protease immobilized onto Ac–HCMSS–NH_2_ nanospheres can be attributed to maintaining the active structural conformation of the enzyme through the multipoint covalent attachment to the matrix and entrapment of the protease molecules within the nanospaces of the hollow core of Ac–HCMSS–NH_2_ nanospheres. Generally, the biotechnological applications of various enzymes could be significantly increased by their utilization in organic media rather than in their natural aqueous conditions. In the last decade the utility of various proteases in organic solvents gained much importance owing to potential applications of proteases in synthesis of ester and peptides in non-aqueous media [32].

**Table 3:** Influence of some inhibitors on the catalytic activity of free and immobilized NPST-AK15 protease. The standard deviations were in the range of 1.5%–3.2%.

Inhibitor	Concentration	Free Enzyme	Immobilized Enzyme
**None**		100	100
**β-mercaptoethanol**	1	94.3 ± 2.4	94.0
	5	88.0 ± 2.2	89.0
**PMSF**	1	15.5 ± 0.16	92.9
	5	0	86.3
**DTT**	1	90.2 ± 1.9	101.8
	5	22.60 ± 0.8	97.5
**EDTA**	2	62.2 ± 2.1	95.0
	5	30.1 ± 1.5	81.5

#### 2.5.4. Influence of Surfactants and Commercial Laundry Detergents

**Figure 10:**
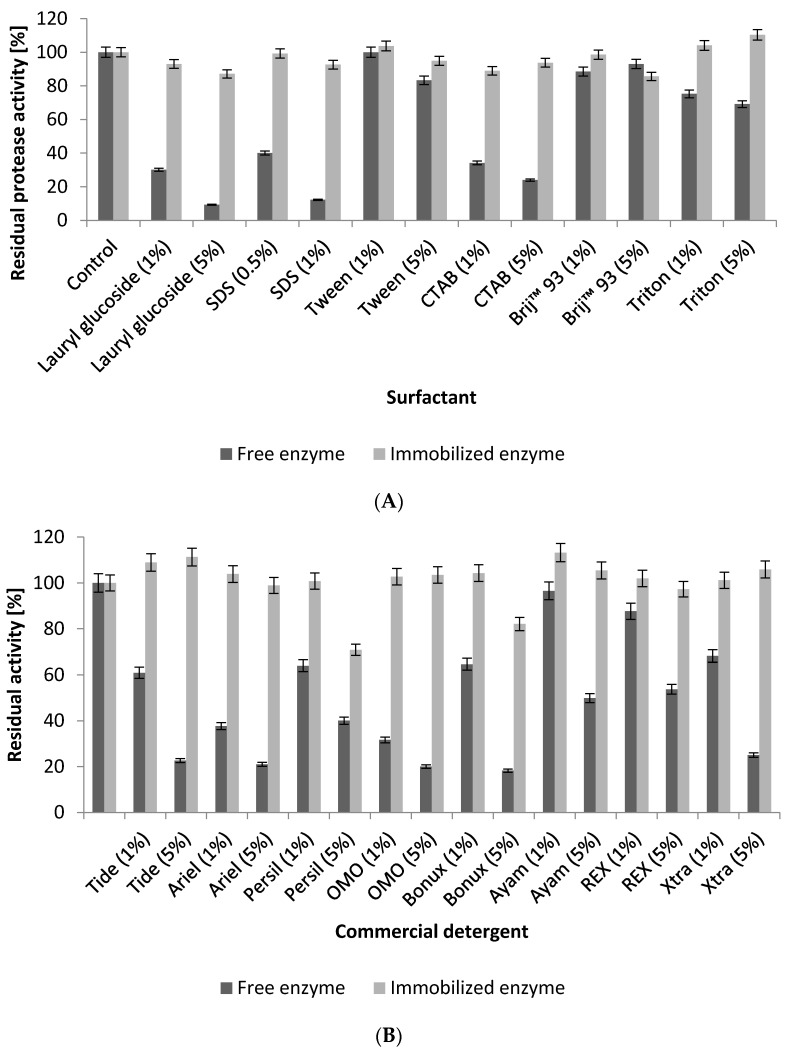
Effect of surfactants (**A**) and commercial detergents (**B**) on stability of free and immobilized NPST-AK15 protease stability. SDS: sodium dodecyl sulfate; CTAB: cetyltrimethylammonium bromide.

The results presented in Figure 10A show of the effect of some surfactants on the stability of free and immobilized NPST-AK15 alkaline protease. It was found that the stability of the immobilized protease exhibited higher stability in 5% lauryl glucoside, sodium dodecyl sulfate (SDS), cetyltrimethylammonium bromide (CTAB) and Triton X-100 by 9.4-, 7.5-, 3.4-, and 1.6-fold, respectively, in comparison to free enzyme. In addition, the stability of immobilized protease in Tween was improved by about 1.1-fold. Finally, to assess the compatibility and feasibility of the enzyme immobilized onto Ac–HCMSS–NH_2_ nanospheres as laundry detergents additive, its stability toward some commercial laundry detergents was investigated. The results illustrated in Figure 10B revealed that the immobilized protease was stable and compatible in most tested commercial laundry detergents. In comparison to free protease, the immobilized enzyme exhibited higher stability in 5% of OMO, Tide, Ariel, Bonux and X-TRA by about 5.2-, 4.9-, 4.7-, 4.5-, and 4.2-fold respectively. In addition, the stability of immobilized protease in 5% Ayam, Persil, and REX as improved by about 2.1-, 1.8-, and 1.8-fold, respectively. The noteworthy stability of NPST-AK15 alkaline protease covalently immobilized on activated Ac–HCMSS–NH_2_ nanospheres in various commercial laundry detergents suggested the effectiveness of the developed nanobiocatalyst as a candidate for detergents formulation.

#### 2.5.5. Operational Stability of Immobilized Protease

**Figure 11:**
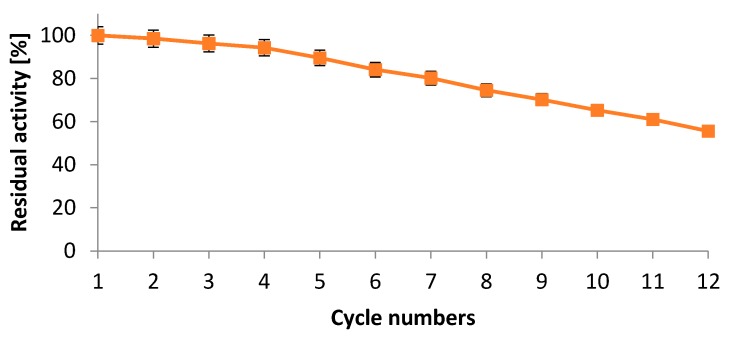
Reusability of NPST-AK15 protease immobilized within hollow core-mesoporous shell silica (HCMSS–NH_2_) nanospheres.

The operational stability of immobilized biocatalysts is considered as one of the most important feature that affects their applications in various bioprocesses. Generally, enzyme leakage from the carriers and enzyme inactivation represent one of the main obstacles for immobilized enzyme applications particularly in repeated batch and continuous process [33]. The reuse of NPST-AK15 protease immobilized onto Ac–HCMSS–NH_2_ nanospheres was studied in several batches for substrate hydrolysis that performed at 40 °C and pH 11. The results indicated that after reutilizations for ten and twelve repeated cycles, the immobilized protease could retain up 65.3% and 55.6% of its initial activity, respectively, revealing a good operational stability of the developed nanobiocatalyst (Figure 11). The operational stability of immobilized biocatalyst is an essential factor for cost-effective application of immobilized enzyme system and development of novel biotechnology.

## 3. Experimental Section

All chemicals were of the analytical grade and, unless specified, obtained from Sigma Chemical Co. (St. Louis, MO, USA).

### 3.1. Alkaline Protease Production and Purification

Alkaliphilic alkaline protease producing *Bacillus* sp. strain NPST-AK15 was previously isolated from Wadi El-Natrun valley hypersaline soda lakes (Wadi El-Natrun, Egypt) [16,17]. The enzyme production was previously optimized [16] and the alkaline protease was purified and characterized [17]. The enzyme production was carried out in production medium of following composition (pH 10.5): yeast extract (7.5 g/L), fructose (20 g/L), K_2_HPO_4_ (1.0 g/L), Mg_2_SO_4_·7H_2_O (0.2 g/L), NaCl (50 g/L), and Na_2_CO_3_ (10 g/L). An overnight culture grown at 40 °C and 150 rpm was inoculated into 500-mL Erlenmeyer flasks containing 100 mL of the production medium and incubated for about 36 h at 40 °C. After the incubation period, cell-free supernatant was recovered by centrifugation at 10,000 rpm for 10 min at 4 °C and assayed for protease activity and protein content. Thereafter, NPST-AK15 protease was purified from cell-free supernatant by combination of ammonium sulfate precipitation and anion exchange column chromatography as we described in previous work [17].

### 3.2. Fabrication of Hollow Core-Mesoporous Shell Silica Nanospheres

#### 3.2.1. Amino-Functionalized Hollow Core-Mesoporous Shell Silica Nanospheres

Amino-functionalized hollow core-mesoporous shell silica (HCMSS–NH_2_) nanospheres were synthesized according to previously reported methods with some modifications [34,35]. Briefly, 4 mL 0.1 M HCl was added to 35 mL of *N*-lauroylsarcosine sodium solution (1 mM) prepared in deionized water, stirred for 1 h and then 0.1 mL of 3-aminopropyltrimethoxysilane (APMS) was added to the solution with further stirring for 10 min. Thereafter 1.5 mL of tetraethyl orthosilicate (TEOS) was added to the reaction, stirred for 10 min, subjected to ultrasonic waves for 4 min at 750 W, and 20 KHz (Sonic vibro cell, Connecticut, CT, USA), and then kept to relax for 1 h. The mixture was heated for 18 h at 80 °C and the obtained solid materials was collected by centrifugation, washed with deionized water and dried for 12 h at 60 °C. The template was removed by acid extraction to keep the hollow core-mesoporous shell silica spheres functionalized with the amino moieties. For removal of anionic surfactant, the final material was suspended in 100 mL of ammonium acetate solution (8%) prepared in ethanol:H_2_O (4:1) and refluxed for 12 h at 90 °C. In order to remove amino groups from the formed hollow core-mesoporous shell silica spheres (HCMSS–NH_2_) to convert it to non-functionalized form, HCMSS–NH_2_ were calcined for 6 h in air stream at 550 °C to give HCMSS nanospheres.

#### 3.2.2. Hydrophobic Ethane-Functionalized Hollow Core-Mesoporous Shell Silica Nanospheres

Ethane-functionalized hollow core-mesoporous shell silica (HCMSS–C_2_H_5_) nanospheres were prepared using post-synthesis functionalization approach according to the method of Brunel [36]. Briefly, 1 mL of 1,2-bis(trimethoxysilyl)ethane (BTME) was added to suspensions of freshly evacuated HCMSS dispersed in toluene (2 g/34 mL), and heated at 120 °C with stirring for 3 h. The solid materials were collected by filtration and washed twice with a diethyl ether–dichloromethane mixture (1:1).

### 3.3. Characterization of the Synthesized Mesoporous Silica Based Nanospheres

A JEOL JSM-2100F electron microscope (Tokyo, Japan) operated at 200 kV was used to obtain Transmission electron microscopy (TEM) images of the synthesized nanomaterials. A Quantachrome NOVA 4200 analyzer (Boynton Beach, FL, USA) was used for measurement of Nitrogen sorption isotherms at 77 K for various nanospheres, which were degassed in a vacuum for 18 h at 200 °C before the measurements. The specific surface area of the nanospheres was calculated using the Brunauer-Emmett-Teller (BET) method at relative pressure range of 0.02–0.20. The pore volumes and size distributions of different silica nanospheres were derived from the adsorption branches of isotherms using the Barrett–Joyner–Halenda (BJH) model. In addition, the total pore volumes (*V*_t_) were calculated from the adsorbed amount at a relative pressure (P/P_0_) of 0.995. Finally, Bruker Vertex-80 spectrometer (Bruker, Massachusetts, MA, USA) was used for recording Fourier transform infrared (FT-IR) spectra.

### 3.4. Alkaline Protease Immobilization

#### 3.4.1. Physical Adsorption

NPST-AK15 protease immobilization by physical adsorption was carried using HCMSS-non, HCMSS–C_2_H_5_ and HCMSS–NH_2_ according to Ibrahim *et al.* [37]. Briefly, 50 mg of carriers were dispersed in 1 mL of glycine buffer (50 mM, pH 8) that contained the purified NPST-AK15 protease and the suspensions were kept for overnight at 4 °C with gentle shaking. The HCMSS–enzyme nanoparticles were recovered by centrifugation for 10 min at 10,000 rpm, and washed twice with glycine buffer to remove any unbound enzyme. The wash solution was collected for protein content and protease activity measurement.

#### 3.4.2. Covalent Attachment

Alkaline protease was covalently immobilized onto amino-functionalized hollow core-mesoporous shell silica (HCMSS–NH_2_) nanospheres according to [38] with some modifications. The matrix was first activated by glutaraldehyde (OCHCH_2_CH_2_CHO) as bifunctional cross linking agent and then conjugation of NPST-AK15 protease to the activated nanospheres. Briefly, 50 mg of the HCMSS–NH_2_ nanospheres were dispersed in 5 mL of glutaraldehyde solution (5%, *v*/*v*), prepared in deionized water and stirred for 2 h at room temperature. The activated matrix (Ac–HCMSS–NH_2_) was recovered from the suspension by centrifugation, and washed with large amounts of deionized water to remove any excess glutaraldehyde. Then, the Ac–HCMSS–NH_2_ nanopartciles (50 mg) was suspended in 1 mL of glycine buffer (50 mM, pH 8) containing the enzyme and maintained overnight at 4 °C with gentle shaking. The Ac–HCMSS–NH_2_–protease nanospheres were recovered by centrifugation at 10,000 rpm for 10 min and washed twice with the same buffer to remove any unbound enzyme. The wash solution was collected for protease activity protein content determination.

### 3.5. Assay of Alkaline Protease Activity

The catalytic activity of soluble and immobilized NPST-AK15 protease was determined using casein as a substrate according to previous method reported with some modifications [23]. A 1.0 mL of casein solution prepared in glycine buffer (50 mM, pH 10.0) was pre-incubated for 5 min at 50 °C, then 1.0 mL of diluted free protease or 1.0 mL glycine buffer containing 50 mg of immobilized enzyme was added and the reaction mixtures were incubated min at 50 °C for 20. Thereafter, the enzymatic reaction was terminated by addition of 1 mL of 10% (*w*/*v*) of trichloroacetic acid (TCA). The mixture was kept at room temperature for 20 min and centrifuged at 10,000 rpm for 10 min to recover the precipitated materials. Lowry method was used to estimate the soluble materials in the mixture supernatant using tyrosine as a standard [39]. One unit of protease activity was defined as amount of enzyme required to liberate 1 µg of tyrosine/min under the experimental assay conditions. Bradford method was used to measure the protein by using bovine serum albumin (BSA) as a standard protein [40].

### 3.6. Properties of Immobilized Protease

#### 3.6.1. Influence of Temperature on Protease Activity and Stability

The optimum temperature of catalytic activity for soluble and immobilized NPST-AK15 protease were determined by measuring the enzyme activity at various temperatures ranged from 30–70 °C under standard assay conditions. The relative activities were calculated as percentage (%) of maximum enzyme activity and the values were plotted against the respective temperature. The thermal stability of both free and immobilized protease was investigated by calculating the residual activity after incubation of enzymes at various temperatures (40–60 °C) for different incubation times (20–120 min). The activity of untreated protease was taken as control (%).

#### 3.6.2. Influence of pH on Protease Activity

The effect of pH on the catalytic activity of both soluble and immobilized NPST-AK15 protease was investigated by assaying the enzyme activity at different pH and appropriate buffers (50 mM), such as sodium acetate (pH 5.0 and 6.0), Tris–HCl (pH 7.0 and 8.0), glycine–NaOH buffer (pH range 9.0–10.5) and carbonate–bicarbonate buffer (pH 11.0–13.0). The substrate solution (1% casein) was prepared in the respective pH buffers and the enzyme preparations (soluble and immobilized) were incubated for 20 min at the optimum temperature of enzyme activity. The relative activities as percentage of maximum activity were calculated and plotted against the respective pH.

#### 3.6.3. Influence of Solvents and Inhibitors

The effects of several enzyme inhibitors on the activity of free and immobilized proteases were studied. The free and immobilized protease was pre-incubated with each inhibitor at final concentrations of 1.0 or 5.0 mM for 30 min at room temperature, and the residual protease activities were determined under the standard assay conditions. The tested inhibitors included ethylene diaminetetraacetic acid (EDTA), β-mercaptoethanol (β-ME), phenylmethanesulfonyl fluoride (PMSF), and dithiothreitol (DTT). For investigation of the stability of free NPST-AK15 protease in the presence of organic solvents, the enzyme samples were pre-treated in various organic solvents (isopropanol, ethanol, methanol, dimethyl ether, diethyl ether, benzene, toluene, chloroform, acetone, butanol, and amyl alcohol) at final concentrations of 25% (*v*/*v*) for 1 h at 40 °C. Thereafter, the residual protease activities were measured. The protease activity assayed in absence of inhibitor or solvents was used as a control (%).

#### 3.6.4. Influence of Surfactants and Commercial Detergents

The stability of soluble and immobilized NPST-AK15 protease was further investigated in the presence of several surfactants such as lauryl glucoside, sodium dodecyl sulfate (SDS), Tween 80, cetyltrimethylammonium bromide (CTAB), Brij^®^93 and Triton X-100. The free and immobilized enzyme was pre-incubated with various surfactants at final concentrations of 1.0% and 5.0% for 1 h at 40 °C, and then the residual enzyme activities were determined. Furthermore, in order to assess the compatibility and feasibility of the immobilized proteases as laundry detergent additives, the enzyme was mixed with various commercial laundry detergent solution including Persil, Ariel, Tide, Bonux, X-TRA, REX, OMO, and Ayam prepared in tap water, with final concentrations of 1% (*w*/*v*) and incubated for 24 h at room temperature. The endogenous proteases in the commercial laundry detergents were inactivated by heating the diluted detergents for 10 min in a boiling water bath before the addition of the NPST-AK15 protease. The protease activity measured in the absence of any detergents or surfactants was used as a control.

#### 3.6.5. Determination of Kinetic Parameters

For estimation of the maximum reaction rate (*V*_max_) and the Michaelis–Menten constant (*K*_m_) of free and immobilized protease, the activity assay was performed using different casein concentrations from 0.1 to 10 mg/mL in 50 mM gylcine buffer at their optimum pH and temperature for soluble and immobilized enzyme. The activity assays were performed as stated above, and then *K*_m_ and *V*_max_ were determined using Lineweaver–Burk plot [41]. In addition, the value of the turnover number (*k*_cat_) which defined as the maximum number of chemical conversions of substrate molecules per second that a single catalytic site will execute for a given enzyme concentration, was calculated from the equation: *k*_cat_ = *V*_max_/[E], where [E] is the enzyme molar concentration in the reaction mixture and *V*_max_ is the maximal reaction rate. All calculated parameters were the mean of triplicate determinations from three independent assays.

#### 3.6.6. Reusability of the Immobilized NPST-AK15 Protease

For investigation of the reusability of immobilized NPST-AK15 protease, the immobilized enzyme was reused for 12 consecutive cycles using 2 mL of the standard reaction mixture. The reaction mixture that contained the immobilized enzyme was incubated in a shaking water bath (120 rpm) at 40 °C for 20 min. At the end of each cycle, the immobilized protease was removed from the reaction medium, washed with glycine buffer and was used to start a cycle using fresh substrate solution.

### 3.7. Statistical Analysis

Experimental results were given as mean value ± SD of three parallel measurements. All statistical analyses were conducted using Microsoft Excel.

## 4. Conclusions

Alkaline protease from *Bacillus* sp. NPST-AK15 was immobilized onto functionalized and non-functionalized HCMSS nanospheres by physical and covalent immobilization. However, NPST-AK15 protease immobilization by covalent attachment onto activated amino-functionalized hollow core-mesoporous shell silica (Ac–HCMSS–NH_2_) nanospheres, through cross linking agent, was more effective showing much higher immobilization yield and loading efficiency in comparison with the physical adsorption approach. The immobilized NPST-AK15 protease exhibited significant improvement of thermal and pH stability in respect to free enzyme. Moreover, NPST-AK15 protease immobilization led to enzyme protection against several inhibitors. Interestingly, the immobilized protease exhibited significant improvement of protease stability in a variety of organic solvents, surfactants and commercial laundry detergents. In addition, the immobilized enzyme exhibited good operational stability up to twelve reaction batches. The developed immobilized alkaline protease is a promising nanobiocatalyst for laundry detergent formulations and various applications for alkaline protease.
